# Relationship Between Steroid Hormone Profile and Premenstrual Syndrome in Women Consulting for Infertility or Recurrent Miscarriage

**DOI:** 10.1007/s43032-023-01375-w

**Published:** 2023-10-18

**Authors:** Joseph V. Turner, Lucas A. McLindon, Damien V. Turner, Yolaine Alefsen, René Ecochard

**Affiliations:** 1 School of Rural Medicine, University of New England, Armidale, Australia; 2 Faculty of Medicine, University of Queensland, Brisbane, Australia; 3 Mater Mothers’ Hospital, Brisbane, Australia; 4 WA Health, Perth, Australia; 5 GHU, Paris Psychiatrie & Neurosciences, Paris, France; 6 Université de Paris Cité, Paris, France; 7 CHU de Lyon, Lyon, France; 8 Université, Claude Bernard Lyon 1, Lyon, France

**Keywords:** Premenstrual syndrome, Premenstrual dysphoric disorder, PMS, PMDD, Progesterone, Oestradiol, Luteal phase

## Abstract

To determine the relationships between luteal-phase steroidal hormonal profile and PMS for a large number of women attending a dedicated fertility clinic. This was a retrospective cross-sectional study on women attending a hospital-based clinic for fertility concerns and/or recurrent miscarriage. All participants were assessed with a women’s health questionnaire which also included evaluation of premenstrual symptoms. Day of ovulation was identified based on the peak mucus symptom assessed by the woman after instruction in a fertility awareness-based method (FABM). This enabled reliable timing of luteal-phase serum hormone levels to be taken and analysed. Between 2011 and 2021, 894 of the 2666 women undertaking the women’s health assessment had at least one evaluable serum luteal hormone test. Serum progesterone levels were up to 10 nmol/L lower for symptomatic women compared with asymptomatic women. This difference was statistically significant (*p* < 0.05) for the majority of PMS symptoms at ≥ 9 days after the peak mucus symptom. A similar trend was observed for oestradiol but differences were generally not statistically significant. ROC curves demonstrated that steroid levels during the luteal phase were not discriminating in identifying the presence of PMS symptoms. Blood levels for progesterone were lower throughout the luteal phase in women with PMS, with the greatest effect seen late in the luteal phase.

**Supplementary Information:**

The online version contains supplementary material available at 10.1007/s43032-023-01375-w.

## Introduction

The neuroendocrine system, as well as progesterone and its metabolites [1], has been implicated in the development of psychiatric conditions such as mood, anxiety and cognitive disorders [2]. It is hypothesised that the cyclic variation of the hypothalamic-pituitary–gonadal (HPG) axis across the menstrual cycle alters the sensitivity of neurotransmitter systems and the function of neural circuits, which may then correspond to the occurrence of psychiatric symptoms in reproductive-aged women [2].

The link between serum progesterone levels and premenstrual syndrome (PMS) remains very much debated. Although varying levels of ovarian hormones are intricately associated with the physiological processes of the menstrual cycle, their role in the precipitation of premenstrual syndrome (PMS) which occurs in luteal phase of the menstrual cycle remains unclear. Some studies suggest that women with and without PMS do not differ in gonadal steroid production [3], but this position is not held by all [4]. Plasma progesterone levels are usually at the forefront of discussions about the link between female steroid hormones and premenstrual syndrome. However, fluctuation of progesterone levels, particularly its fall at the end of the luteal phase, or an imbalance in the progesterone/oestradiol ratio has sometimes been correlated with PMS. Studies in premenstrual dysphoric disorder (PMDD) investigating the relationship between severity of symptoms and plasma concentration of progesterone have provided conflicting results [5–12]. Additional studies have also been conducted on allopregnanolone, a progesterone metabolite, with one recent report suggesting an inverted U-shaped relationship between severity of negative mood symptoms and allopregnanolone serum concentration [13]. Establishing whether there is an identifiable steroid hormone profile in women with PMS is an important step in clarifying this issue.

There is limited evidence regarding the relationship between PMS and luteal-phase serum concentration of steroid hormones. Reports in the literature have only involved small numbers of symptomatic participants, with results based on studies of less than 50 women having been published previously [3–12, 14].

Identifying the day of ovulation allows clinicians and researchers to correctly time the taking of blood samples from the woman for specific days in the luteal phase of her menstrual cycle. Knowledge of timing of blood hormone measurement is essential since steroid hormone levels vary throughout the menstrual cycle. Definitive timing of ovulation is best given by serial peri-ovulatory ultrasound scanning. However, this is not practical for a large sample of women, with or without PMS.

A validated clinical practice is the use of the peak mucus symptom to approximate the day of ovulation [15]. This physiological sign identifies ovulation within 2 days and can be observed by a large number of women in a home-based setting [16, 17]. Practitioners instructing in one of the methods of fertility awareness, for example, the Billings Ovulation Method, Creighton Model FertilityCare System or Symptothermal Method (STM) routinely teach women to track mucus and other vaginal discharge patterns during their menstrual cycle. Accurate recording of ovulation provides insights into other health-related matters. This may include undertaking selective intercourse for achieving or avoiding pregnancy, identifying vaginal and cervical inflammatory conditions and characterising ovulatory disorders such as polycystic ovary syndrome (PCOS) [18–20].

Hormonal dysregulation in the luteal phase may contribute to infertility or recurrent miscarriage. Thus, women presenting for investigation of infertility can have targeted luteal-phase hormonal assessment, with accurate menstrual cycle charting. A post-ovulatory hormonal profile is commenced on the third day after the peak mucus sign is identified, followed by repeat serum progesterone and oestradiol determinations on the fifth, seventh, ninth and eleventh days after the peak sign [21].

A complete work-up in this population should entail enquiry into the symptoms of PMS and/or PMDD, which occur in the luteal phase of a menstrual cycle [22]. In the present study conducted at a dedicated fertility clinic, the objective was to establish relationships between luteal-phase steroidal hormonal profile and PMS for a large number of women.

## Materials and Methods

### Study Population

In the present retrospective cross-sectional study, the women included were those who were at least 18 years of age and who received a comprehensive clinical assessment at the Natural Fertility Service of the Mater Mothers’ Hospital (MMH), South Brisbane, Australia, for pre-existing fertility concerns (subfertility/infertility) and miscarriage history. Women were referred to the Natural Fertility Service by their General Practitioner, specialist Obstetrician & Gynaecologist, or were self-referred. Exclusion criteria covered women who were subfertile due to tubal obstruction, who had breastfed in the last 12 months, were currently using contraception, were currently pregnant and were currently using any prescribed fertility-enhancing medications or supplements, including vitex agnus.

The clinical assessment included a women’s health questionnaire, which included reproductive and gynaecological history, medical and mental health. This incorporated the presence of premenstrual symptoms of significance. All women recorded their fertile cycles using the SymptoThermal method of recording [23].

Women freely consented to the use of their data for approved research including publication of results. Data was collected during the period 2011 to 2021 with approval from the Mater Misericordiae Human Research Ethics Committee (approval number: ERM 72620, amendment AM/MML/72620 V1).

### PMS Assessment

Women in the study were asked by self-questionnaire about the presence or absence of PMDD symptoms listed in the DSM-5 [24]. To be included, these symptoms had to occur for 4 or more days before the onset of menstrual bleeding.

These symptoms are as follows: Four mood symptoms: “Mood swings, cry easily”; “Irritability, anger”; “Depression, hopelessness”; “Anxiety, feeling wired on edge”. Seven other: “Less interest in usual activities”; “Difficulty with concentration”; “Fatigue, lack of energy”; “Change in appetite, cravings”; “Difficulty sleeping, too much sleep”; “Loss of control, overwhelmed”; “Physical symptoms such as breast tenderness, bloating, weight gain, headache”.

### Identification of the Peak Day for Ovulation

The women in this study were taught to identify the Peak day of cervical mucus, which corresponds closely to the time of ovulation [25]. During the fertile window, the cervix responds to increasing oestradiol levels by increasing the production of oestrogenic-type mucus. Mucus changes from a thicker appearance and a damp or wet sensation to a slippery or lubricative sensation and/or thin and stretchy appearance. The peak sign, as noted by the woman, is indicated on the last day of this fertile mucus discharge at the vulva [15]. Data was collected from spontaneous ovulation cycles.

### Hormonal Steroid Levels

Luteal-phase plasma oestradiol and progesterone determinations were routinely undertaken. Samples were drawn at local Mater Pathology collection centres. All specimens initially were analysed using the Siemens Immulite® immunoassay system. After 12 June 2012, the Abbott Architect® automated immunoassay was used and all results were multiplied by 1.08 to correct for the measured bias found in this laboratory between the Immulite and Architect methods. Oestradiol was expressed in picomole per litre, and progesterone was expressed in nanomole per litre. The samples were taken on one or more of the following days in the luteal phase: days 3, 5, 7, 9, 11 after the peak day.

We did not test for differences in the levels of these hormones on different days of the luteal phase. The differences exist and have been documented elsewhere [26].

### Luteal-Phase Hormonal Profiles

The luteal phase of each of the participants was classified according to the type of possible impairment following the following criteria: type 1: short luteal phase, < 8 days, last progesterone is < 6.9 nmol/L; type 2: sub-optimal profile, 9–16 days long, peak < 29 nmol/L; type 3: late luteal defect, 9–16 days long, abrupt drop of progesterone (post-peak + 9) is ≤ 50% of (post-peak + 7); type 4: early luteal defect, 9–16 days, slow rising oestradiol and progesterone, peak at/after peak + 9; type 5: isolated luteal oestradiol deficiency, normal progesterone profile; “Anovulation”: peak progesterone < 22 nmol/L [21]; pregnant in work-up.

### Statistical Methods

Patient characteristics, including age and BMI, were assigned classes, for which means and distribution were calculated.

The pregnancy history of participants included in the study was tabulated and presented as percentages.

The number of symptoms presented by the participants, as well as the frequency of each symptom, was counted and percentages were calculated.

The distribution of the types of luteal-phase hormonal profiles was calculated in the absence of symptoms and in the presence of each of them, whether alone or associated with other symptoms.

The distributions of age and BMI in women with no symptoms and those with at least one symptom were calculated and compared using Student *t* tests.

The type of luteal phase observed in the presence of each symptom was then compared to that of women without symptoms by a logistic regression (likelihood ratio test).

The mean of the progesterone, oestradiol and progesterone/oestradiol ratio levels and its 95% confidence interval was then calculated and tabulated. Then, the differences between these averages observed in the participants without symptoms versus those with each of the symptoms were calculated and tested by an analysis of variance. These differences were presented graphically for ease of reading.

Finally, using ROC curves, we investigated the extent to which the level of hormonal steroids can act as a test for the presence of at least one PMS symptom. Hormone level, successively progesterone, oestradiol and their ratio, was considered the diagnostic method, and sick/not sick was the presence/absence of at least one PMS symptom. We calculated the areas under the ROC curve, with the 95% confidence interval obtained by bootstrap, and presented the curves.

There were very few missing values for the variables studied. Wherever appropriate, we gave the number of women for whom the information was available.

Statistical calculations and graphs were made with the R software and the significance threshold was set at 0.05.

## Results

**Fig. 1:**
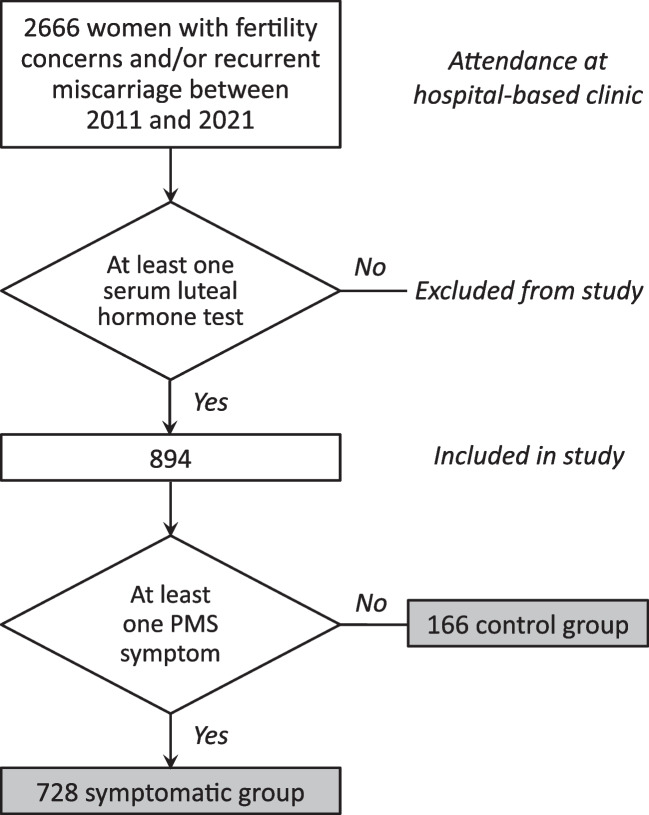
Study population selection flow diagram

Between January 2011 and December 2021, a total of 2666 women sequentially presenting at the Natural Fertility Service received the women’s health and reproductive assessment for inclusion in the current study. Of these, 894 had had at least one serum hormonal test on a known specific day following the peak day (Fig. 1).

### Characteristics and Pregnancy History of Participants Included in the Study

The age and body mass index of the participants are presented (Table 1) and pregnancy history (Table 2). Nearly 40% of the study population were 35 years old or older, with a similar number in the overweight or obese range (BMI ≥ 25 kg/m^2^). Sixty percent of women had had at least one pregnancy, while nearly half of the women had also had at least one miscarriage. The presence or absence of at least one symptom was not significantly related to age or BMI.

**Table 1:** Age and body mass index of the participants included in the study (*N* = 894)

Characteristic	*N* (%)	Control i.e. no symptoms (%)	Women with ≥ 1 symptom (%)	*p* value^a^
Age (years)				
18–24	32 (4)	7 (5)	25 (3)	0.353
25–34	487 (54)	75 (53)	412 (57)	
35–40	235 (26)	33 (23)	202 (28)	
> 40	107 (12)	27 (19)	80 (11)	
Missing	33 (4)			
Body mass index (kg/m^2^)				
Underweight (< 18.5)	31 (3)	4 (4)	27 (4)	0.101
Normal weight (18.5–24.9)	447 (50)	60 (57)	387 (54)	
Overweight (25–29.9)	220 (25)	34 (32)	186 (26)	
Obese (≥ 30)	128 (14)	8 (8)	120 (17)	
Missing	68 (8)			

^a^Student *t* test

**Table 2:** Pregnancy history (*N* = 894), number of women and row percentages

Characteristic	None *N* (%)	One *N* (%)	Two *N* (%)	Three *N* (%)	Four *N* (%)	≥ Five *N* (%)
Gravidity (*n* = 882^a^)	327 (37)	152 (17)	130 (15)	104 (12)	69 (8)	100 (11)
Parity (*n* = 880)	582 (66)	226 (26)	44 (5)	19 (2)	5 (1)	4 (0)
Miscarriages (*n* = 881)	466 (53)	123 (14)	119 (14)	102 (12)	37 (4)	34 (4)

^a^*n* = number of women for whom information is available

#### PMS

Considering the number of symptoms of PMS experienced by each participant, of the 894 women with measured serum ovarian hormone values, 166 (18.6%) did not have any symptoms of PMS (Fig. 1). Women having one symptom (102, 11.4%), two symptoms (209, 23.4%) and three symptoms (102, 11.4%) made up nearly half the cohort. There was a diminishing number of women experiencing increasing numbers of symptoms of PMS, with 7 (< 1%) experiencing all 11 symptoms.

The most frequent symptoms experienced by women were *Physical symptoms such as breast tenderness, bloating, weight gain and headache* (626 over 894, i.e. 70%), with the least frequent symptoms being *Less interest in usual activities* (24, 2.7%) and *Difficulty with concentration* (27, 3%).

### PMS and Luteal-Phase Hormonal Profiles

Table 3 presents the distribution of luteal-phase profiles excluding 52 participants out of 894 (6%), for whom the cycle was a pregnancy cycle (50), a short cycle (1) or a cycle for which this classification was not made (1).

**Table 3:** PMS symptoms and luteal-phase hormonal profiles

Symptom	*N* (row %)	Anovulation *N* (%)	Early luteal defect *N* (%)	Late luteal defect *N* (%)	Normal *N* (%)	Sub-optimal profile *N* (%)	*p* value^a^
Control (no symptom)	166 (100)	18 (11.9)	13 (8.6)	34 (22.5)	68 (45)	18 (11.9)	
Mood swings, cry easily	256 (100)	35 (13.7)	29 (11.3)	59 (23)	94 (36.7)	39 (15.2)	0.505
Irritability, anger	421 (100)	57 (13.5)	47 (11.2)	112 (26.6)	144 (34.2)	61 (14.5)	0.230
Depression, hopelessness	136 (100)	19 (14)	13 (9.6)	41 (30.1)	41 (30.1)	22 (16.2)	0.133
Anxiety, feeling wired on edge	107 (100)	15 (14)	9 (8.4)	36 (33.6)	30 (28)	17 (15.9)	0.070
Less interest in usual activities	23 (100)	5 (21.7)	1 (4.3)	8 (34.8)	2 (8.7)	7 (30.4)	0.003
Difficulty with concentration	26 (100)	7 (26.9)	1 (3.8)	8 (30.8)	4 (15.4)	6 (23.1)	0.017
Fatigue, lack of energy	308 (100)	41 (13.3)	34 (11)	82 (26.6)	110 (35.7)	41 (13.3)	0.432
Change in appetite, cravings	235 (100)	22 (9.4)	28 (11.9)	63 (26.8)	81 (34.5)	41 (17.4)	0.150
Difficulty sleeping, too much sleep	98 (100)	8 (8.2)	8 (8.2)	32 (32.7)	32 (32.7)	18 (18.4)	0.131
Loss of control, overwhelmed	49 (100)	6 (12.2)	4 (8.2)	14 (28.6)	14 (28.6)	11 (22.4)	0.217
Physical symptoms such as breast tenderness, bloating, weight gain, headache	595 (100)	75 (12.6)	58 (9.7)	156 (26.2)	219 (36.8)	87 (14.6)	0.468

^a^Likelihood ratio test comparing women without symptoms to those with symptoms

There were 842 participants who had available luteal-phase hormonal profiles, of whom 166 were in the symptom-free (control) group. Forty-one percent (68 of 166) of these had normal luteal-phase hormonal profiles, and the rest were distributed among the various categories of abnormalities.

The symptoms for which the distribution differed significantly from that of controls were *Less interest in usual activities* (*p* = 0.0019) and *Difficulty with concentration* (*p* = 0.0117). Less than 20% of these women had a normal luteal-phase hormonal profile. About a quarter of them had an anovulation-type profile, i.e. a very low progesterone level (less than 22 nmol/L).

### PMS and Luteal-Phase Steroid Hormones

Supplementary Tables 1, 2 and 3 respectively present the averages of progesterone, oestradiol and progesterone/oestradiol ratio observed in participants without symptoms and in the presence of symptoms.

Progesterone levels in symptomatic individuals are on average lower than in non-symptomatic individuals. The difference is at most 10 nmol/L, which represents a decrease of about 30%.

Although not statistically significant, oestradiol levels in symptomatic patients are generally lower on average than in non-symptomatic patients. The difference is at most 70 pmol/L, which represents a decrease of about 15%.

The ratio of progesterone-to-oestradiol in symptomatic patients is on average lower than in non-symptomatic patients. The difference is at most 2, which represents a decrease of about 22%.

**Fig. 2:**
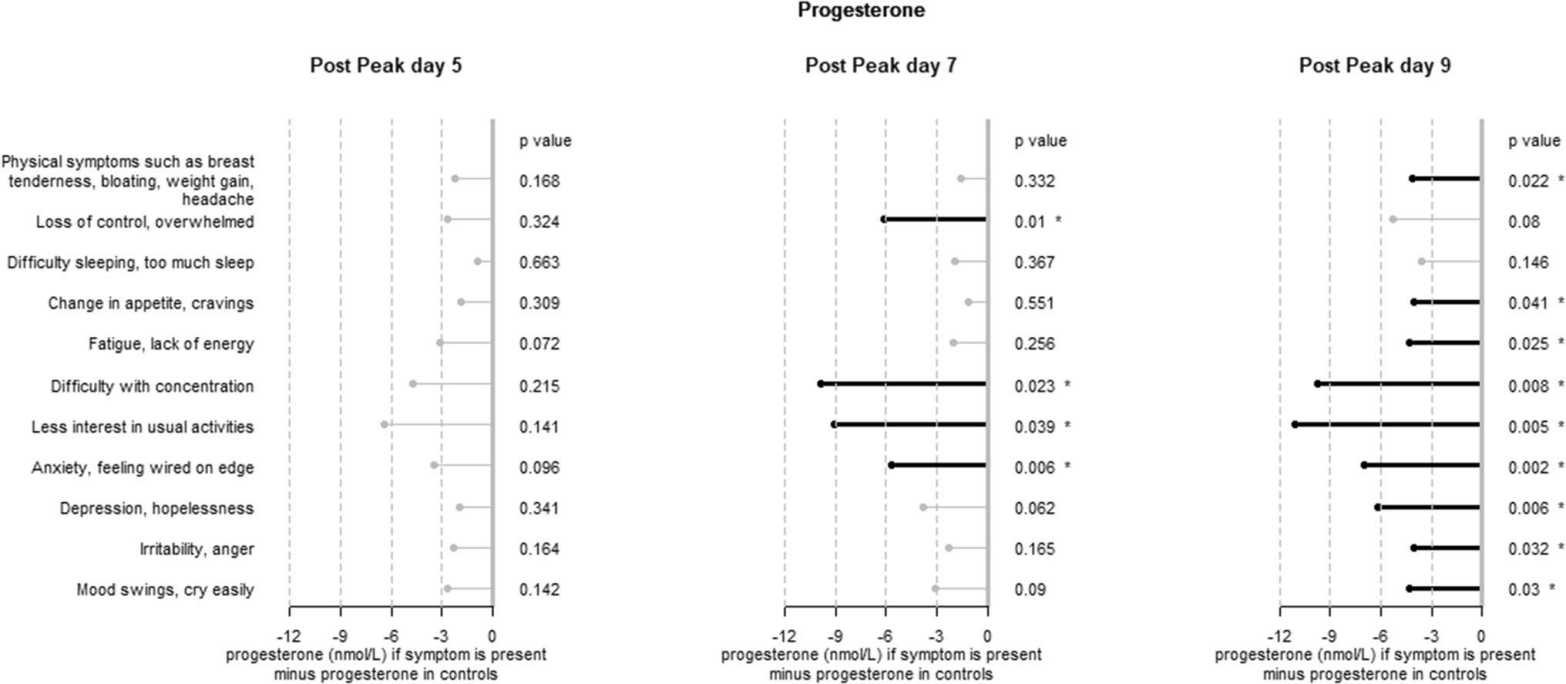
Difference in plasma concentration between controls (166 women reporting no symptoms) and women with one or more symptoms. Progesterone. A star * indicates a statistically significant result

**Fig. 3:**
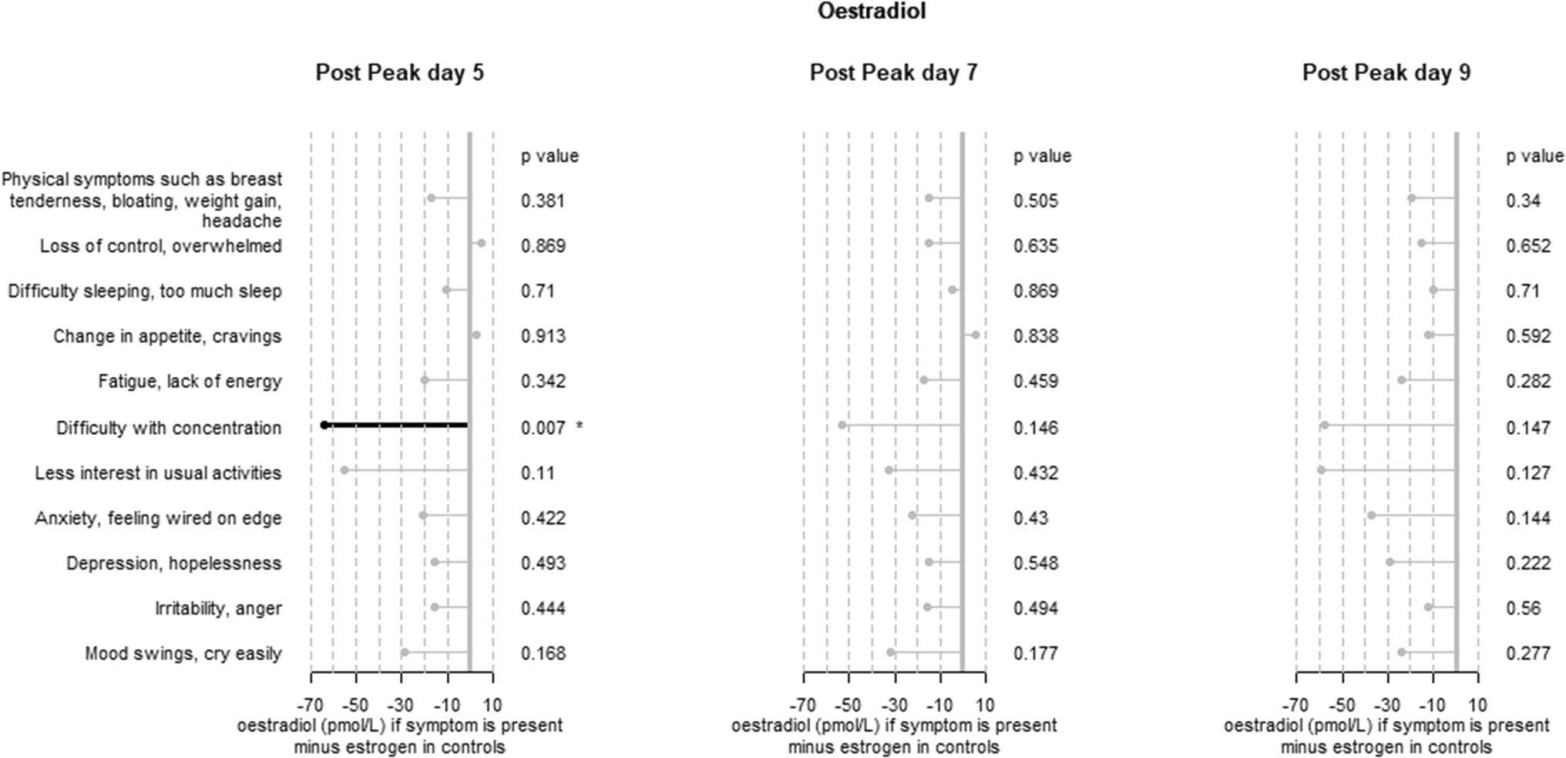
Difference in plasma concentration between controls (166 women reporting no symptoms) and women with one or more symptoms. Oestradiol. A star * indicates a statistically significant result

**Fig. 4:**
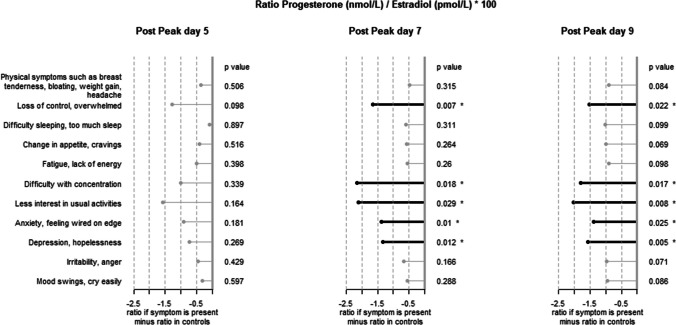
Difference in plasma concentration between controls (166 women reporting no symptoms) and women with one or more symptoms. Ratio progesterone/oestradiol. A star * indicates a statistically significant result

Figures 2, 3 and 4 show directly the differences between these averages in the presence of the symptoms in comparison with the averages observed in the controls. It is mainly at peak + 9 that a large number of significant differences were observed for progesterone: the only symptoms that were not significantly associated with low progesterone at peak + 9 were *Loss of control overwhelmed* (but the difference was significant at peak + 7) and *Difficulty sleeping, too much sleep.*

Except *Change in appetite, cravings and Loss of control, overwhelmed*, all other symptoms were associated with lower oestradiol levels than in women without symptoms. But only one difference was statistically significant: at peak + 5, in case of *Difficulty with concentration.*

Five symptoms were associated with significantly lower progesterone-to-oestradiol ratios than in symptom-free participants at peak + 7 and peak + 9. These were *Loss of control*, *overwhelmed*, *Difficulty with concentration*, *Less interest in usual activities*, *Anxiety*, *feeling wired on edge* and *Depression*, *hopelessness*.

### ROC Curves

**Fig. 5:**
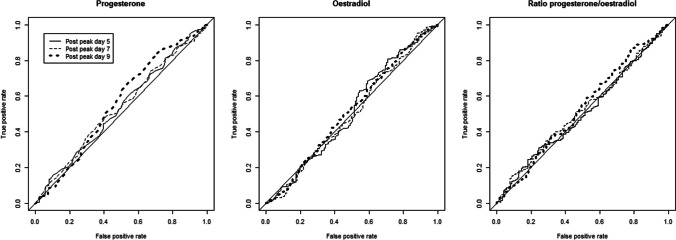
Hormone level, successively progesterone, oestradiol and their ratio, was considered the diagnostic method and sick/not sick was the presence/absence of at least one PMS symptom

Figure 5 shows that steroid levels during the luteal phase are not discriminating in identifying the presence of one or more symptoms of PMS. Supplementary Table 4 presents the areas under the curve which are very close to 0.5, i.e. low, expressing the lack of discrimination.

## Discussion

This study found that among women consulting for infertility or recurrent miscarriage, blood levels of progesterone and oestradiol were on average lower throughout the luteal phase in women with PMS than in those without.

This was larger and more consistently statistically significant for progesterone than for oestradiol. In particular, the difference in progesterone was more significant 9 days, or beyond, after ovulation.

The progesterone-to-oestradiol ratio was lower in women with PMS symptoms than in women without symptoms. Nevertheless, there was no evidence that the ratio would be more strongly related to symptomatology than the progesterone level itself.

Despite the clear association between low progesterone, particularly at or beyond 9 days after ovulation, and the presence of PMS symptoms, low progesterone was not a reliable predictor of PMS. This was shown by the low area under the curve of the ROC curves: symptoms may be absent in a woman with low progesterone, and progesterone may be normal in a woman with PMS. Lower levels of oestradiol were seen in women with PMS symptoms; however, this difference in oestradiol levels was mostly not statistically significant.

The measurement of progesterone on specific days after ovulation as timed by the peak mucus symptom in a large number of women gives a higher level of evidence than work done previously on only a small number of women. Our results invite reconsideration of a widespread opinion that there is no causal link between low progesterone levels and risk of premenstrual syndrome [27]. However, it would be useful to verify whether what we have observed in women consulting for infertility or recurrent miscarriage also occurs outside this clinical context.

The lower progesterone and oestradiol levels at peak + 5, peak + 7 and peak + 9 seen in the present study reflect lower average ovarian (corpora lutea) activity in women with PMS than in women without PMS among women consulting for infertility or recurrent miscarriages.

Progesterone was on average 30% lower in women with PMS than in those without. However, the progesterone measurement was not discriminating in identifying women with PMS. This means that low progesterone levels in some women are not consistent with PMS. There have been previous studies investigating treatment of PMS with progesterone. These have found no benefit of progesterone; however, they did not select for women with existing low serum progesterone levels [28–30]. In light of results from the present study, it may be important to study the benefit of progesterone support as a treatment for PMS, specifically in the subpopulation of women with low plasma progesterone levels beyond day 9 after peak.

The study of the pathophysiology of PMS could benefit from the results presented here. How changes in sex steroids influence PMS symptoms remains to be understood. Allopregnanolone, an anxiolytic metabolite of progesterone, which is known to have a cerebral effect, may be at physiologically ineffective levels in women with low plasma progesterone levels [31, 32]. Allopregnanolone is a modulator of the GABA receptor, enhancing the effect of γ-aminobutyric acid (GABA). It has been suggested that the drop in progesterone at the end of the luteal phase thus causes changes in the effects of central nervous system neurotransmitters such as γ-aminobutyric acid (GABA) [33]. Elsewhere, it has been proposed that a drop in endorphin is one of the primum movens [34]. In a study, PMS patients showed a decrease in plasma beta-endorphin in the week preceding menses and during the first days of menstrual flow [35].

Our results invite the question, *what predominates in women with PMS: the deficit of sex steroid secretion or endorphin production?* If the latter, this would explain the discordant results of many studies, and that, in our data, low progesterone levels were not present in all women with PMS. To clarify this point, it would be necessary to have a sufficient number of women with progesterone and beta-endorphin levels measured a fixed number of days after ovulation. Further study would then test whether a progesterone intake or a treatment aimed at increasing beta-endorphins may assist particular subgroups of women with significant PMS. These hypotheses would be compatible with the uniformly recognised benefit of serotonergic treatments for PMDD.

One limitation in this study is the specific population studied, being women consulting for infertility or recurrent miscarriage. The conclusions cannot be extended to the general population without caution. The high prevalence of PMS in our sample may be due to the link between PMS and infertility, which has already been observed [36].

A further limitation is the self-reporting of symptoms and duration using patient recall. An attempt to mitigate this effect involved a clinician review to clarify the symptoms at the consultation. Future studies could include prospective collection of this data to improve reliability.

## Conclusion

This study found that among women consulting for infertility or recurrent miscarriage, blood levels of progesterone and oestradiol were on average lower throughout the luteal phase in women with PMS than in those without PMS.

This was larger and more consistently significant statistically for progesterone than for oestradiol and more significant 9 days or more after ovulation as identified by the peak symptom. Nevertheless, progesterone cannot be proposed as a biological test for the diagnosis of PMS in an infertility population. A low progesterone could be a marker of a sub-type of PMS; however, this postulation requires further investigation.

### Supplementary Information

Below is the link to the electronic supplementary material.

Supplementary file1 (DOCX 25 KB)

## Data Availability

The data that support the findings of this study are available from the corresponding author, JT, upon reasonable request.
