# Supplementary material for: Relationship Between Steroid Hormone Profile and Premenstrual Syndrome in Women Consulting for Infertility or Recurrent Miscarriage
Source: Reprod Sci. 2023 Oct 18;31(3):736–45. doi: 10.1007/s43032-023-01375-w (PMC10912418; doi:10.1007/s43032-023-01375-w)
Supplement: Supplementary file 1 — Supplementary file1 (DOCX 25 KB) [file 43032_2023_1375_MOESM1_ESM.docx]

**Supplementary Table 1.** Mean plasma progesterone level (nmol/L) in controls and in women with the symptom

| Subgroup | Post-peak day 5 | Post-peak day 7 | Post-peak day 9 |
| --- | --- | --- | --- |
| Controls (no symptom) | 36.4 (33.6 ; 39.3)^§^ | 37.4 (34.7 ; 40.2) | 32.0 (28.8 ; 35.2) |
| Mood swings, cry easily | 33.8 (31.7 ; 35.9) | 34.4 (32.1 ; 36.6) | 27.7 (25.5 ; 29.9) |
| Irritability, anger | 34.1 (32.4 ; 35.8) | 35.1 (33.4 ; 36.9) | 28.0 (26.3 ; 29.7) |
| Depression, hopelessness | 34.4 (31.5 ; 37.4) | 33.6 (30.7 ; 36.5) | 25.8 (22.8 ; 28.8) |
| Anxiety, feeling wired on edge | 32.9 (29.9 ; 36.0) | 31.7 (28.8 ; 34.7) | 25.0 (22.0 ; 28.0) |
| Less interest in usual activities | 30.0 (21.6 ; 38.3) | 28.4 (20.3 ; 36.5) | 20.9 (14.1 ; 27.6) |
| Difficulty with concentration | 31.7 (24.5 ; 38.9) | 27.6 (19.6 ; 35.5) | 22.2 (15.9 ; 28.6) |
| Fatigue, lack of energy | 33.3 (31.4 ; 35.2) | 35.4 (33.4 ; 37.5) | 27.7 (25.7 ; 29.7) |
| Change in appetite, cravings | 34.6 (32.4 ; 36.8) | 36.3 (33.9 ; 38.7) | 27.9 (25.8 ; 30.1) |
| Difficulty sleeping, too much sleep | 35.5 (32.4 ; 38.6) | 35.5 (32.3 ; 38.7) | 28.4 (24.7 ; 32.1) |
| Loss of control, overwhelmed | 33.7 (29.1 ; 38.4) | 31.3 (27.4 ; 35.1) | 26.7 (21.6 ; 31.8) |
| Physical symptoms such as breast tenderness, bloating, weight gain, headache | 34.2 (32.8 ; 35.6) | 35.9 (34.4 ; 37.3) | 27.9 (26.5 ; 29.3) |

^§^ average (95% confidence interval)

**Supplementary Table 2.** Mean plasma oestradiol level (pmol/L) in controls and in women with the symptom

| Subgroup | Post-peak day 5 | Post-peak day 7 | Post-peak day 9 |
| --- | --- | --- | --- |
| Controls (no symptom) | 447.8 (413.9 ; 481.7) ^§^ | 478.8 (438.7 ; 518.9) | 440.6 (403.8 ; 477.5) |
| Mood swings, cry easily | 419.1 (396.0 ; 442.2) | 446.7 (422.4 ; 471.0) | 416.2 (391.8 ; 440.7) |
| Irritability, anger | 432.6 (412.9 ; 452.3) | 463.3 (443.5 ; 483.1) | 428.4 (409.4 ; 447.3) |
| Depression, hopelessness | 432.5 (404.0 ; 460.9) | 464.0 (436.5 ; 491.5) | 411.4 (381.9 ; 440.9) |
| Anxiety, feeling wired on edge | 427.0 (388.5 ; 465.5) | 456.6 (418.1 ; 495.1) | 403.1 (368.3 ; 437.8) |
| Less interest in usual activities | 392.8 (332.4 ; 453.2) | 446.1 (371.0 ; 521.3) | 381.4 (312.0 ; 450.8) |
| Difficulty with concentration | 383.8 (351.6 ; 416.1) | 425.4 (363.3 ; 487.6) | 382.7 (311.2 ; 454.2) |
| Fatigue, lack of energy | 427.9 (404.1 ; 451.7) | 461.5 (439.0 ; 484.0) | 416.8 (393.6 ; 440.0) |
| Change in appetite, cravings | 450.3 (421.2 ; 479.4) | 484.0 (453.4 ; 514.6) | 428.3 (401.8 ; 454.7) |
| Difficulty sleeping, too much sleep | 437.3 (392.8 ; 481.8) | 474.0 (432.8 ; 515.1) | 430.4 (390.2 ; 470.5) |
| Loss of control, overwhelmed | 452.6 (405.4 ; 499.7) | 463.9 (416.0 ; 511.8) | 425.7 (370.7 ; 480.6) |
| Physical symptoms such as breast tenderness, bloating, weight gain, headache | 431.2 (415.2 ; 447.2) | 464.1 (447.6 ; 480.6) | 421.2 (405.4 ; 437.0) |

^§^ average (95% confidence interval)

**Supplementary Table 3.** The average ratio of progesterone (nmol/L) to estradiol (pmol/cl) in controls and in women with the symptom

| Subgroup | Post-peak day 5 | Post-peak day 7 | Post-peak day 9 |
| --- | --- | --- | --- |
| Controls (no symptom) | 9.1 (8.1 ; 10.1)^§^ | 8.7 (7.8 ; 9.6) | 7.7 (6.7 ; 8.7) |
| Mood swings, cry easily | 8.8 (8.1 ; 9.4) | 8.2 (7.6 ; 8.7) | 6.8 (6.3 ; 7.2) |
| Irritability, anger | 8.6 (8.2 ; 9.1) | 8.0 (7.6 ; 8.5) | 6.7 (6.3 ; 7.2) |
| Depression, hopelessness | 8.4 (7.6 ; 9.2) | 7.4 (6.8 ; 8.0) | 6.1 (5.6 ; 6.7) |
| Anxiety, feeling wired on edge | 8.2 (7.3 ; 9.0) | 7.3 (6.7 ; 7.9) | 6.3 (5.6 ; 7.1) |
| Less interest in usual activities | 7.5 (5.5 ; 9.6) | 6.6 (4.9 ; 8.3) | 5.7 (4.5 ; 6.8) |
| Difficulty with concentration | 8.1 (6.3 ; 9.9) | 6.5 (5.1 ; 8.1) | 5.9 (4.8 ; 7.1) |
| Fatigue, lack of energy | 8.6 (8.0 ; 9.2) | 8.1 (7.7 ; 8.6) | 6.8 (6.4 ; 7.2) |
| Change in appetite, cravings | 8.7 (8.0 ; 9.4) | 8.1 (7.6 ; 8.7) | 6.7 (6.3 ; 7.2) |
| Difficulty sleeping, too much sleep | 9.0 (8.0 ; 10.0) | 8.1 (7.3 ; 8.9) | 6.7 (6.0 ; 7.4) |
| Loss of control, overwhelmed | 7.8 (6.7 ; 9.0) | 7.1 (6.2 ; 7.9) | 6.2 (5.3 ; 7.1) |
| Physical symptoms such as breast tenderness, bloating, weight gain, headache | 8.7 (8.3 ; 9.1) | 8.2 (7.9 ; 8.6) | 6.8 (6.5 ; 7.1) |

^§^ average (95% confidence interval)

**Supplementary Table 4.** Area under curve (95% confidence intervals)

Hormone level - successively progesterone, oestradiol and there ratio, was considered the diagnostic method and sick/not sick was presence/absence of at least one PMS symptom

|  | Post-peak day 5 | Post-peak day 7 | Post-peak day 9 |
| --- | --- | --- | --- |
| Progesterone | 0.53 (0.48,0.59) | 0.54 (0.48,0.59) | 0.56 (0.5,0.61) |
| Oestradiol | 0.52 (0.45,0.57) | 0.50 (0.44,0.56) | 0.52 (0.46,0.58) |
| Progesterone/oestradiol | 0.51 (0.45,0.56) | 0.52 (0.47,0.57) | 0.53 (0.48,0.58) |
